# The safe implementation of a prison-based methadone maintenance programme: 7 year time-series analysis of primary care prescribing data

**DOI:** 10.1186/1471-2296-15-64

**Published:** 2014-04-08

**Authors:** Nat MJ Wright, Charlotte French, Victoria Allgar

**Affiliations:** 1 HMP Leeds Healthcare Department, 2 Gloucester Terrace, Armley, Leeds LS12 2TJ, England; 2 University of York, John Hughlings, Jackson Building Heslington, York YO10 5DD, England

**Keywords:** Methadone maintenance, Prison medicine, Opiates, Dependence

## Abstract

**Background:**

Internationally there is policy support for the introduction of methadone maintenance programmes into prison settings. Increasingly GPs are encouraged to undertake this work although concerns remain regarding the safety of such programmes. This study sought to evaluate the impact and safety of the introduction of a general practitioner with a special interest (GPsi) in substance misuse led methadone prescribing service into a UK prison between 2003 and 2010.

**Methods:**

Time series analysis of secondary prescribing data pertaining to opiate maintenance therapies, opiate detoxification therapies and opiate related deaths for the time period 2003 to 2010.

**Results:**

Results show that following introduction of a GPsi in substance misuse there was a statistically significant increase in both methadone maintenance and detoxification treatments. Over time the rate of methadone maintenance prescribing plateaued with a corresponding decrease in the rate of methadone detoxification prescribing. There were no methadone related deaths in prison over the study period.

**Conclusion:**

The phased introduction of opiate replacement therapies into a busy remand prison did not result in any deaths within the prison for which opiate replacement was identified as the cause. GPsi led opiate prescribing programmes can be introduced safely into secure environments.

## Background

Recently published statistics show that the prison population for England and Wales was 84,586 at the end of August 2010 [1]. This figure represents an increase in the prison population of approximately 30 per cent since 1999. The increase was due to a combination of tougher sentencing and enforcement outcomes, and a more serious mix of offence groups coming before the courts. It equates to a prison population rate of 153/100,000 of the national population. Internationally there are countries with a higher proportion of the population imprisoned. For example the United States has the highest prison population rate in the world with 714 per 100,000 of the national population in prison and Russia 629/100,000 of the national population, [2].

Upon reception into prison up to 80% of offenders test positive for either heroin or cocaine [3]. A cross-sectional survey of a sample of the UK prison population conducted in 1998 revealed that 38% of male remand prisoners and 48% of sentenced prisoners admitted to using drugs during their current period of imprisonment. The survey was conducted at a time when there was very little drug treatment available in the UK prison setting. The practice of coerced detoxification from opiate maintenance was commonplace upon reception into prison leading to a class action brought against the Home Office for negligent drug treatment provision whilst in prison [4]. An out of court compensation settlement led to a growing pressure to develop prison based drug treatment services. Compensation was provided as it was acknowledged that opiate maintenance and detoxification therapies had significant potential to reduce harms associated with illicit drug withdrawal. There was an established evidence base that opiate maintenance reduced rates of crime, illicit drug use, and injecting practice yet prisoners were not routinely provided this treatment. Also there was an emerging UK evidence base that rates of self-harm and suicides were markedly increased in the week following reception into prison and that uncontrolled symptoms of opiate withdrawal were a contributory factor [5]. At that time the vast majority of primary care services were provided by doctors employed by the Home Office and not the National Health Service (NHS). Not all practitioners offering primary care services held a certificate of accreditation in general practice.

Nevertheless some drug users were able to become abstinent whilst in prison. However in the absence of safe prison based primary care prescribing of opiate maintenance there is an increased risk of drug related death upon release from prison. This is due to accidental overdose in the community if similar drug misuse practices are re-commenced upon release following a period of enforced abstinence in prison leading to a loss of tolerance to prescribed opiates. An analysis of almost 50,000 newly released prisoners in England and Wales during the period 1998 to 2000 highlighted over 440 deaths, of which over 250 (59%) were drug-related [6]. In the year following release, the drug-related mortality rate was 5.2 per 1000 per annum among men and 5.9 per 1000 per annum among women. However, all-cause mortality in the first and second weeks following prison release for men was 37 and 26 deaths per 1000 per annum, respectively. In total, 95% of these deaths were drug-related. All-cause mortality in the first and second weeks following prison release for women was 47 and 38 deaths per 1000 per annum, respectively, all of which were drug-related [6]. These data highlights the period of the first two weeks post-release from prison as a time of high risk for opioid-related deaths.

In June 2004, in such a context of litigation and drug related death post prison-release, an opiate maintenance programme was introduced into a remand prison in the North of England with a population of approximately 1200 prisoners. The introduction was phased and preceded the widespread introduction of such programmes across both the national and international prison estate. Examples of the international context at that time are that in the Australian penal system, a prison based randomised controlled trial evaluating the effectiveness of methadone maintenance had just been published [7]. Key findings were that at five-month follow-up in prison, those receiving methadone maintenance reported significant reductions in heroin use, drug injection and syringe sharing. Four years later, a USA randomised controlled trial evaluating the effectiveness of prison based opiate maintenance therapy was published. The researchers randomly allocated two hundred and eleven users prisoners to either counselling in prison, with passive referral to treatment upon release; counselling and transfer (counselling in prison with transfer to methadone maintenance treatment upon release); or counselling and methadone (methadone maintenance and counselling in prison, continued in a community-based methadone maintenance program upon release). Key findings were at one-month follow-up post release a higher engagement in treatment and lower proportion of individuals testing positive for illicit opiates in the group who received prison based methadone maintenance [8]. However neither of these two trials specifically mentioned GP involvement in the provision of opiate maintenance to prisoner populations. We are unaware of any published empirical research pertaining to core competencies required by GPs in prescribing that has traditionally been viewed as high risk. In response to such a gap in the evidence base this paper reports the findings from a time series analysis of seven years prescribing data for opiate maintenance which took place at a time of increasing international research activity evaluating the introduction of such maintenance therapy into prison settings.

## Methods

The introduction of a methadone maintenance programme was undertaken by a general practitioner with special interest (GPsi) in substance misuse who commenced prison based drug treatment in December 2003 in Her Majesty’s Prison (HMP) Leeds, a remand prison with a capacity for 1283 prisoners, where the average length of stay for each prisoner was thirteen weeks. A consensus document between the UK Royal College of General Practitioners (RCGP) and Royal College of Psychiatrists, supported by the National Treatment Agency for Substance Misuse defined the competencies of a GPsi as having received specific higher-level training in the management of substance misusers in primary care, usually the Royal College of General Practitioners’ Certificate in the management of drug misuse in primary care, Part 2. The document recommended that such practitioners can deliver a fuller range of drug treatment services and, as a result of additional ongoing higher-level training and professional development activity, are able to work more autonomously, accepting referrals from generic GP colleagues, and take responsibility for more complex cases in substance misuse [9].

For the first six months from January to June 2004 methadone was not prescribed but organisational changes were made to facilitate the introduction of a methadone maintenance programme. Such changes included writing a policy containing guidelines for safe prescribing, dispensing and administration; and stock requisition. In June 2004 the GPsi commenced a weekly prescribing clinic for opiate maintenance. Fifteen months later the number of clinics was increased to five per week. All new treatment inductions (new commencements) were seen in this clinic. However over the following two years patients stable on methadone maintenance treatment were followed up in generic (ie not substance misuse specific) prison GP clinics. Over time new inductions (commencements) onto maintenance treatment and methadone detoxification regimes were initiated in clinics run by prison GPs who had acquired core competencies to work as a GPsi in substance misuse. “New treatment inductions” included those who were initiated on methadone for the first time following a clinical history consistent with opioid dependence and a urine sample positive for either opiates, methadone or buprenorphine. It included new treatment inductions in either normal prison working hours or evening clinics for patients newly received into prison. It also included those assessed in such evening clinics where community prescribed opiate maintenance had been disrupted due to an absence of methadone prescribing in police custody at the time the study was taking place. Repeat treatment inductions were excluded from analysis. Such patients included those who were re-imprisoned during the study period who had previously been included as a “new treatment induction”. Methadone prescribing for both maintenance and detoxification was initiated at a dose of 30 mg daily if the patient was assessed on first night reception, and at a dose of 20 mg daily if the patient was assessed in the routine prison clinic. The rationale for such prescribing regimes was that compared to patients presenting in routine clinics, patients presenting at first night prison reception typically both reported and exhibited more severe symptoms and signs of withdrawal. Subsequently for those receiving a detoxification regime at an initial dose of 20 mg od (once daily), the dose would be increased the following day to 30 mg od for a period of six days prior to a reducing regime from 30 mg od to zero over a two week period. In total such a prescribing regime would be of three weeks duration. For those receiving a maintenance prescribing regime at an initial dose of 20 mg od, the dose would be increased the following day to 30 mg od. Any subsequent increases would only take place following review by a GP. Subsequently published national guidelines for prison recommend that “to ensure patient safety within this context, methadone treatment programmes should be established through a process of dose induction. Initial doses of five to ten milligrams of methadone (1 mg in 1 ml mixture) are to be given, at least six hours apart” [10]. National guidelines published in 2007 pertaining to drug treatment recommended “in general, the initial daily dose will be in the range of 10–30 mg. If tolerance is low or uncertain then 10–20 mg is more appropriate. With heavily dependent misusers who are tolerant, and where the clinician is experienced or competent, a first dose can be up to 40 mg but it is unwise to exceed this dose” [11].

Anonymised secondary data for both maintenance and detoxification treatments was retrieved from the Pharmacy Manager prison pharmacy dispensing databases for the period covering the start of January 2003 to end March 2010. Once retrieved, the data was aggregated into discrete three month time periods according to the date of first receipt of a methadone prescription. Methadone prescribing was introduced into the prison in May 2004. Therefore by obtaining data at equally- spaced time intervals (ie. three month time periods) both pre and post-intervention, it was possible to analyse by an interrupted time-series methodology. Durbin-Watson statistics were used to test for serial correlation (a relationship between values separated from each other by a given time lag). The trend component of this time-series is examined to determine the long-term variation of the trends in prescribing methadone maintenance and detoxification therapies in the prison and community. The basic model: Yt = bo + b1T + b2D + b3P + et, where T is time from the start of the observational period, D is a dummy variable for pre or post intervention and P is time since the intervention; et is the random variation at time t not explained by the model. Ordinary Least Squares regression was undertaken to analyse prescribing trends over time, with 3 independent variables: T – time, D – dummy for pre-post intervention and P – time since the intervention. The model can determine the change over time before the intervention was implemented, change in the outcome measure from the last time point before the intervention to the first time point after the intervention, and the difference in the slope of the time period before the intervention and the slope of the time period after the intervention. Ordinary least squares regression assumes that the error terms associated with each observation (time point) are uncorrelated. Prior to data extraction we discussed the proposed research with Wales Medical Research Ethics Committee who advised that formal ethical approval was not required as the study was a service evaluation using anonymised aggregated secondary data from a service in which the principal investigator conducted routine clinical work.

## Results

Over the study period a total of 4551 patients were inducted onto methadone maintenance and 3181 patients received a detoxification treatment.

For methadone maintenance, the numbers of individuals receiving maintenance prescriptions rise steadily from its introduction in 2004, to 282 in April - June 2007. Subsequently, prescriptions remain within the range of 245-311 until October - December 2009, where they fall to 216 before rising slightly at the beginning of 2010 to 232.

**Figure 1:**
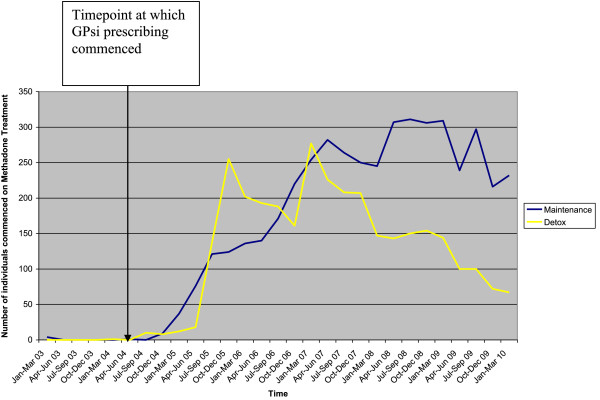
Number of individuals commenced on Methadone for either Maintenance or Detoxification at HMP Leeds from 2003-2010.

Figure 1 shows the number of inductions “new commencements” maintenance and detoxification episodes between the time period start of January 2003 to end of March 2010 and this is displayed in table format in Table 1.

**Table 1:** Numbers of methadone maintenance and detoxification new treatments issued from 2003-2010

**Time period**	**Methadone maintenance new treatment induction episodes**	**Methadone new detoxification treatment episodes**
Jan-Mar 2003	4	0
Apr-Jun 2003	0	0
Jul-Sep 2003	0	0
Oct-Dec 2003	0	0
Jan-Mar 2004	0	1
Apr-Jun 2004	1	0
Jul-Sep 2004	0	10
Oct-Dec 2004	9	8
Jan-Mar 2005	37	12
Apr-Jun 2005	76	18
Jul-Sep 2005	121	138
Oct-Dec 2005	124	255
Jan-Mar 2006	136	202
Apr-Jun 2006	140	193
Jul-Sep 2006	171	188
Oct-Dec 2006	220	161
Jan-Mar 2007	254	277
Apr-Jun 2007	282	226
Jul-Sep 2007	264	208
Oct-Dec 2007	250	207
Jan-Mar 2008	245	147
Apr-Jun 2008	307	143
Jul-Sep 2008	311	150
Oct-Dec 2008	306	154
Jan-Mar 2009	309	144
Apr-Jun 2009	239	100
Jul-Sep 2009	297	100
Oct-Dec 2009	216	72
Jan-Mar 2010	232	67
**TOTAL**	**4551**	**3181**

Ordinary least squares regression was undertaken to analyse prescribing trends over time. Durbin-Watson statistics to test for serial correlation of the error terms was not significant (p = 0.556), meaning that there was no autocorrelation. The trend prior to intervention was flat (p = .978), as would be expected. Following the intervention of GPsi prescribing there was an increase in patients receiving maintenance (Graph 1), but this was not significant (p = 0.085). Also there was no significant change in trend immediately after the intervention (0.494). However over time post the intervention of a GPsi there is a statistically significant increase for methadone maintenance treatment episodes (p = 0.002).

As regards the results for detoxification treatments, prior to the time-point April - July 2006, detoxification treatments appear to have been preferred to maintenance. The number of detoxification prescriptions rose dramatically from 18 (April-June 2005) to 255 (October – December 2005), staying higher than those for methadone until the end of 2006, when they fell to 161. There then appears to be a rapid increase in detoxification therapies prescribed in January-March 2007, reaching 277. However after this increase, methadone detoxification prescriptions have remained lower than those for methadone maintenance and have decreased steadily from 277 at the beginning of 2007 to 67 in January – March 2010. Ordinary least squares regression was undertaken to analyse prescribing trends over time. Durbin-Watson statistics to test for serial correlation of the error terms was not significant (p = 0.597), meaning that there was no autocorrelation. The trend prior to intervention was flat (p = 0.935), as would be expected. Following the intervention of GPsi prescribing there was an increase in patients receiving detoxification (Graph 1), which was significant (p = 0.016). There was no significant change in trend after the intervention (p = 0.864). However over time post the intervention of a GPsi there was no statistically significant increase in detoxification treatment episodes (p = 0.619).

### Drug-related deaths

There were no opioid related deaths in the prison over the study period prior to the introduction of methadone maintenance treatment. During the time period of 2003-2010 there was one death of a patient receiving methadone treatment at the time of death. His death occurred in 2006 one week after initiation of methadone at a daily dose of 30 mg. However toxicology investigations highlighted the illicit use of mirtazapine, a sedative antidepressant [12]. At inquest the coroner reported that the prescribing of methadone maintenance was appropriate in this case and therefore, this patient’s death was not attributed to his methadone treatment. Thus, there are no methadone related deaths recorded at HMP Leeds since its introduction in 2004.

## Discussion

Following the intervention of a GPsi led prescribing service for substance misuse there was a steady increase over the following four and half years in patients being initiated onto methadone maintenance. The fact that the increase only became statistically significant over time is evidence that the implementation was phased to ensure that the system was safe. From the time-point April-June 2008 a further increase in the number of patients being initiated onto methadone maintenance is evidence of a second prison based GPsi receiving the core competencies to work with substance misuse and the practice of methadone induction therefore becoming normalised in the prison healthcare department.

Following the introduction of a GPsi led prescribing service there was an immediate statistically significant increase in patients receiving detoxification regimes. However two years after the introduction there was a decline in detoxification prescribing, the decline starting in the time-point April-June 2006. This can be explained by the fact that the local prescribing protocol changed. Initially due to pressure on the service a patient could only be initiated onto opiate maintenance if they had failed to become abstinent upon completion of a detoxification regime. However as over 80% of drug users in the prison failed to achieve abstinence [13], the protocol was changed to permit patients to be inducted onto methadone maintenance where appropriate. In the time-point January -March 2007 there was a further increase in methadone detoxification treatments. This can be explained by the recruitment of a new GP who at the time of recruitment did not possess the core competencies of a GPsi. Therefore the prescribing interventions of the GP were limited to offering detoxification (and not maintenance) regimes. As the GP acquired the core competencies to practice as a GPsi, the number of maintenance treatments continued to rise and over time a corresponding fall in detoxification treatments.

In 2001, a UK based taskforce highlighted that the prison environment can be a difficult and complex one in which to work as a doctor. It highlighted that the nature of the prisoner population meant that prison based doctors would require particular skills in dealing with alcohol and substance misuse. It also highlighted the inherent risks of professional isolation working in prison settings and recommended that the priority should be in developing a robust structure of primary care provision in prison settings [14]. A key clinical governance concern was that not all doctors providing primary care services in the UK prison estate at that time were trained in the skills of general practice. In 2001 the UK Royal College of General Practitioners commenced a training unit dedicated to training general practitioners in the knowledge and skills required to competently offer treatment to drug users. At the time of writing, since the inception of this course 14,137 individuals have been trained in the basics of drug treatment and 2016 have received training to confer the competencies required to practice as a GPsi in substance misuse [15]. We would suggest that the RCGP has learned lessons that can be disseminated internationally. The RCGP through the Membership of Royal College of General Practitioners International academic examination currently has a successful formula for internationally conferring generic primary care core competencies. The challenge for the RCGP will be how it can replicate this successful generic international primary care model in the field of primary care drug treatment. We would suggest that networking with international policy leads, and international marketing of training programmes are key to disseminating best training practice. International data pertaining to the background of doctors providing primary care based drug treatment in prison settings is lacking. It is possible that other countries are facing similar clinical governance concerns regarding the safety of prison primary care based drug treatment. There is evidence from research conducted in the Belgian prison healthcare service of prisoners consulting the GP 3.8 times more than a demographically equivalent population in the community [16]. The high consultation rate was explained in part by health concerns relating to drug misuse. However there is a paucity of research evaluating primary care based drug treatment provision. Clearly if internationally prisoners are high users of primary care services then general practitioners who have received training in the provision of safe drug treatment will be key to increasing the international provision of prison based drug treatment. It would not be our aspiration that such general practitioners would necessarily adopt the UK title of “general practitioner with a special interest”. Rather the purpose of this paper has been in describing the core competencies to ensure safe primary care provision. It is the competencies rather than the terminology that we would wish to share with the international readership.

The main strength of this research is in highlighting that such primary care development is possible and that methadone replacement therapies can safely be introduced into prison settings providing such a practice is by GPs possessing a GPsi level of core competency. Additionally generic GPs are able to safely offer detoxification regimes within clear prescribing protocols. This is an important message to the GP field. The burden of investigation upon GPs practicing in secure environments is intense. In the UK, every death in custody is by statute subject to scrutiny by Coroner’s Inquiry [17] and investigation by the Prisons and Probation Ombudsman [18]. Therefore GPs seeking to provide opiate maintenance therapies in the prison setting should only do so having undertaken either further training or supervision in the management of substance misuse. In 2007, the World Health Organisation through its Health in Prisons Project recommended opiate maintenance therapies are made more widely available across the international prison estate [19]. Our research has provided a primary care workforce model which will be able to facilitate the safe and effective implementation of this key international policy recommendation. A primary care workforce is critical to increasing availability of drug treatment. Indeed in 1978 the WHO endorsed the critical importance of primary care organisations as the central focus for promoting improvements in population health [20].

The main limitation of this research is that it is limited to prescribing data collated at one remand prison and therefore we would be confident that our findings could be generalised with confidence only to remand prisons with similar health needs relating to substance misuse. Also it was not possible to cross-check our data against coronial records to evaluate the impact of the programme upon opioid related deaths post release from prison. We suggest this is an area for future research activity. Also our research did not collect data pertaining to buprenorphine medication as this was not routinely prescribed in the prison at the time of initiating data collection. The reason for this was that the UK prisons at that time had widespread problems regarding buprenorphine abuse and therefore prescribing of such medication was not encouraged [21].

Our research has shown that a GPsi led prison drug treatment service is safe if on first night reception the first induction dose of methadone does not exceed 30 mg and if in routine “outpatient” prison clinics the first induction dose of methadone does not exceed 20 mg. This provides evidence for future reviews of current UK national prison clinical guidelines which currently recommend a more cautious approach of initial induction at doses of methadone 5-10 mg given at least six hours apart” [10]. Not every prison will have access to a GPsi. However increasingly clinical leads in prisons are practicing at a GPsi level of competency and our findings would support higher initial induction doses being prescribed in prison first night reception centres.

In demonstrating that methadone maintenance prescribing can be safely introduced into prison settings, our findings raise questions regarding long-term outcomes of patients receiving such maintenance treatment. The outcomes of survival, re-offending, abstinence and retention in treatment post-release are all areas that merit future research activity. Also comparing prescribing data between prisons internationally would facilitate the comparing of clinical standards and frameworks and lead to enhanced sharing of international best practice. Future research activity should also analyse prescribing data for buprenorphine as this is increasingly being used for both maintenance and detoxification in prison settings.

## Conclusion

In summary our research has highlighted that a GPsi led phased implementation of methadone maintenance into prisons does not compromise patient safety.

## Competing interests

The authors declare that they have no competing interests.

## Authors’ contributions

NW provided access to the prescribing data from HMP Leeds and participated in the design of the project and drafted the manuscript. CF gathered and processed the data required to analyse the number of individuals commenced on methadone and also assisted in drafting the manuscript. VA conducted the statistical analysis and drafted this into the methods and results sections. All authors read and approved the final manuscript.

## Authors’ informations

Nat Wright is the Associate Medical Director for Specialist Services and Vulnerable Groups, Leeds Community Healthcare. This role entails providing medical leadership to a cluster of prisons.

Charlotte French undertook this work whilst completing a BSc in primary health care.

Victoria Allgar is Senior Lecturer in Medical Statistics at Hull and York Medical School/Health Sciences, University of York.

## Pre-publication history

The pre-publication history for this paper can be accessed here:

http://www.biomedcentral.com/1471-2296/15/64/prepub
